# Modeled Population Connectivity across the Hawaiian Archipelago

**DOI:** 10.1371/journal.pone.0167626

**Published:** 2016-12-08

**Authors:** Johanna L. K. Wren, Donald R. Kobayashi, Yanli Jia, Robert J. Toonen

**Affiliations:** 1 Joint Institute for Marine and Atmospheric Research, University of Hawai‘i at Mānoa, Honolulu, Hawai‘i, United States of America; 2 Hawaiʻi Institute of Marine Biology, School of Ocean and Earth Science and Technology, University of Hawaiʻi at Mānoa, Kāne‘ohe, Hawaiʻi, United States of America; 3 Ecosystems and Oceanography Program, Pacific Islands Fisheries Science Center, National Oceanographic and Atmospheric Administration, Honolulu, Hawai‘i, United States of America; 4 International Pacific Research Center, University of Hawaiʻi at Mānoa, Honolulu, Hawai‘i, United States of America; University of California Santa Cruz, UNITED STATES

## Abstract

We present the first comprehensive estimate of connectivity of passive pelagic particles released from coral reef habitat throughout the Hawaiian Archipelago. Potential connectivity is calculated using a Lagrangian particle transport model coupled offline with currents generated by an oceanographic circulation model, MITgcm. The connectivity matrices show a surprising degree of self-recruitment and directional dispersal towards the northwest, from the Main Hawaiian Islands (MHI) to the northwestern Hawaiian Islands (NWHI). We identify three predicted connectivity breaks in the archipelago, that is, areas in the mid and northern part of the archipelago that have limited connections with surrounding islands and reefs. Predicted regions of limited connectivity generally match observed patterns of genetic structure reported for coral reef species in the uninhabited NWHI, but multiple genetic breaks observed in the inhabited MHI are not explained by passive dispersal. The better congruence in our modeling results based on physical transport of passive particles in the low-lying atolls of the uninhabited NWHI, but not in the anthropogenically impacted high islands of the MHI begs the question: what ultimately controls connectivity in this system?

## Introduction

Determining levels and patterns of connectivity is vital for understanding metapopulation dynamics and persistence, and is essential for effective resource management see [1–5]. Over ecological time scales, population persistence depends on either the ability to retain locally produced larvae, i.e. self-recruitment, or the ability to import larvae from nearby areas, i.e. connectivity [6–8]. Self-recruitment is a metric describing how open or closed a population is, which in turn describes its resilience [7,9]. Open populations receive an influx of larvae from outside sources, making them more resilient to local disturbances but limited in potential for local adaptation [10,11]. Closed populations are more sensitive to local disturbances and possess a greater potential for local adaptation since they are dependent on locally produced offspring and have a more direct link between local production and recruitment. Marine population studies have historically worked under the assumption that marine fish populations are open—that is, they receive larvae from other populations some distance away [12] due to the dispersal ability and relatively long larval duration of marine fish larvae. However, studies in recent years have challenged this notion, showing that despite a strong larval dispersal ability many marine reef populations appear closed, with larvae staying “close to home” [6,13–16]. We no longer assume all marine populations to be open, and the focus is now on determining the extent to which marine populations exchange larvae (see [1,2]). Knowing the connectedness of a population is vital in effectively managing the population and designing functioning marine reserves.

Most coastal marine species have a biphasic life cycle, in which dispersal takes place predominantly during the pelagic larval stage of the life cycle [17]. Some species lay benthic eggs that develop into pelagic larvae, whereas others spawn gametes directly into the water column, where they drift as passive particles until they develop swimming abilities similar to benthic hatchlings. Larvae can be feeding or non-feeding in the water column, and the pelagic larval phase may last for minutes to months in the pelagos before they return to the benthos to settle. Each of these life-history differences have predictable impacts on observed population genetic structure [18,19], but the biological and physical factors driving dispersal in the sea are not well understood and difficult to generalize. Factors controlling successful dispersal can be species specific [15,20–22], depend on timing of spawning events [23,24], and vary among locations [25–29].

The Hawaiian Archipelago, located in the subtropical North Pacific Ocean, is a 2,500 km long chain of volcanic islands and atolls, stretching from 19°N in the MHI to 30°N in the NWHI. The Hawaiian Archipelago is one of the most isolated on the planet, and home to one of the largest marine reserves in the world, Papahānaumokuākea Marine National Monument (PMNM). There is a high level of endemism in the Hawaiian Archipelago [30,31], and due to its remote location, has unique management needs [32,33]. While the MHI are populated with active fisheries and heavy anthropogenic loading, the NWHI are uninhabited and fully protected with little anthropogenic influence [34]. One of the hopes for establishing PMNM, which was the largest MPA on the planet at that time, was a spillover effect where the protected fish populations in PMNM would replenish fish stock in the MHI. Unfortunately, this hope has been little supported among studies to date of both invertebrates and fishes [35–38]. The lack of spillover from PMNM to the MHI has been attributed to the prevailing surface currents moving larvae up the island chain from the MHI towards the NWHI [35,39].

Because management needs vary greatly between the heavily populated MHI and the uninhabited PMNM, it is vital that we understand the population dynamics between these areas as well as within them. Well-connected populations with numerous dispersal pathways among sites are more resilient, that is, more likely to recover from disturbance. Conversely, isolated populations that are highly dependent on self-recruitment for population maintenance are less likely to recover after a disturbance and face a greater risk of extinction [3–5,7].

Extensive population genetic work has been done to characterize population structure for fish and invertebrates to infer exchange among sites throughout the Hawaiian Archipelago (reviewed by [20,22]), but only a handful of studies have focused on estimating dispersal during the larval stage [35,36,39–45]. To date, all such studies focus on either a single species of interest, a small region of the archipelago or a very limited time period. Here, we present the first comprehensive dataset describing modeled potential connectivity among sites throughout the entire Hawaiian Archipelago using a biophysical model coupled with eddy resolving ocean currents. We use a purely physical model with passive particles to determine likely patterns of potential connections within the archipelago and Johnston Atoll because detailed information on larval behavior, mortality rates and population sizes are not currently available for the vast majority of species in Hawai‘i. The results from this study will set the groundwork for future studies to use more realistic biophysical models that incorporate such factors as larval behavior as they become available.

## Methods

### Dispersal model

#### MITgcm

The Massachusetts Institute of Technology general circulation model (MITgcm) solves the incompressible Navier-Stokes equations on a sphere in discretized forms employing a finite-volume technique [46]. The regional MITgcm implementation for the Hawaiian Archipelago extends from 175°E to 150°W and from 15°N to 35°N at a 0.04° (~4km in the region) resolution. In the vertical direction, the water depth is divided into 50 layers with a thickness ranging from 5 m near the surface to 510 m near the bottom. It is forced at the surface by winds derived from the Advanced Scatterometer (ASCAT) observations with a 0.25° resolution, and by heat and freshwater fluxes obtained from the European Center for Medium-Range Weather Forecast (ECMWF) Interim Reanalysis at a 1.5° resolution. The ocean state, as estimated by the global HYCOM prediction system at a 0.08° resolution [47], is used to define the initial and open boundary conditions. The simulation period runs from May 2009 to May 2014. We use the flow fields in the 100 m model layer to disperse particles in our Lagrangian tracking experiments (see below), as this layer has shown to be the best predictor of settlement in the region [42,43].

#### Habitat

**Fig 1:**
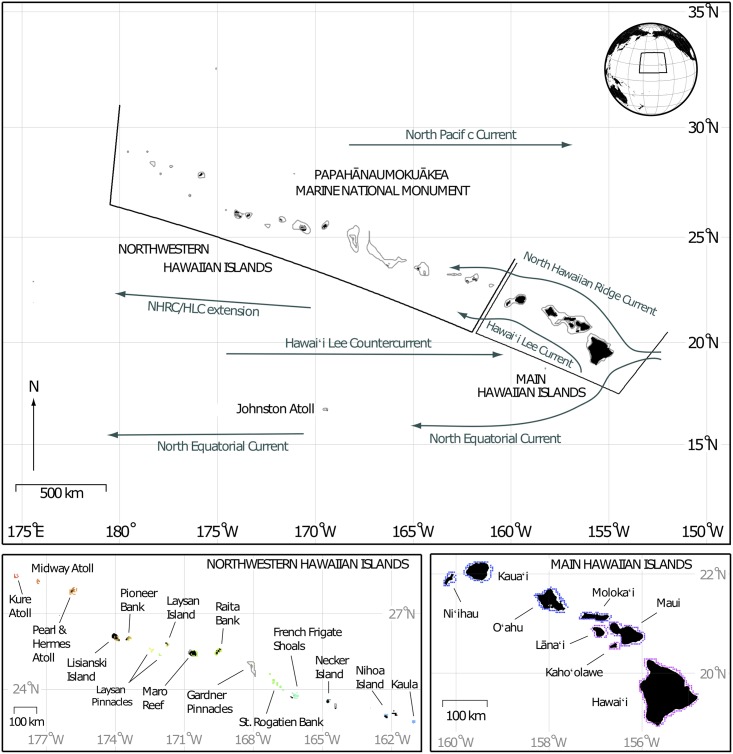
Map of the Hawaiian Archipelago. Top panel showing the Hawaiian Archipelago domain of the dispersal model with the major surface currents (in green) identified (after Lumpkin 1998). Bathymetry lines denote 1000 and 50 m isobaths. Bottom panels show coral reef habitat pixels for the Northwestern Hawaiian Islands and Main Hawaiian Islands respectively, with each island's habitat pixels shown as a separate color. Bathymetry lines in NWHI denote 50m depth isobath.

For this study, we included all available coral reef habitat in the Hawaiian archipelago and Johnston Atoll. Johnston Atoll is the nearest reef to the Hawaiian Archipelago, located 1390 km southwest of the Big Island of Hawai‘i. We chose to include Johnston Atoll in the habitat definition because there are shown biogeographic ties between Johnston Atoll and the Hawaiian Archipelago [41,48–51]. To generate our habitat map, we used habitat defined as ‘coral reef’ in IKONOS-derived data for the Northwest Hawaiian Islands [52,53] and the data set presented in [54] for the MHI, and created a 4-km^2^ grid of that habitat, totaling 687 habitat pixels. The habitat pixels were additionally grouped into 31 different islands/banks/atolls (hereafter referred to as islands) to allow for island scale comparisons (Fig 1).

#### Model initialization

To investigate the exchange of particles among locations in the Hawaiian Archipelago, we used a Lagrangian bio-physical particle tracking model [40,42] coupled with the flow fields from the MITgcm simulation described above. Eddy diffusivity was set to 250 m^2^/sec, consistent with drifters in Hawaiian waters (following [35]). We released 50 particles (virtual larvae) daily from May 2, 2009, until April 10, 2014, from 687 coral reef habitat pixels totaling just over 62 million released particles for each model run. We used a pelagic larval duration (PLD) of 45 days, representative for most reef fish [55]. Previous studies show that PLD’s longer than 45 days do not significantly alter settlement probabilities in the MHI [42]. For a particle to be considered “settled”, it had to be within a 5 km radius of the center of a habitat pixel on the last day of its PLD (on day 45)(Table 1). Preliminary studies show no change in connectivity patterns when particles are allowed to settle across a range of PLD, so we chose a static PLD as opposed to range of PLD’s or a settlement window because we were interested in the physical drivers of dispersal. The dispersal model was run three times and the output averaged for consistency.

**Table 1:** Glossary of often used terms. Terms and definitions used throughout the Hawaiian Archipelago connectivity study.

Potential connectivity	The modeled estimate of connectivity of a site using physical oceanographic attributes and limited biological factors influencing dispersal ability of the particles.
Settlement	In this study we define settlement as any particle within 5km of the center of a habitat pixel on day 45 after release
Self-recruitment	A particle that settles back onto the same island from which it was released
Connectivity break	An area over which few, if any, particles are exchanged
Source-sink index	The ratio of export of particles and import of particles to an area, divided by the total number of successfully transported particles.

To test the robustness of the model with respect to ocean circulation model resolution [56] we ran identical biophysical model runs, forcing the model with current velocities from the global HYCOM at a 0.08° resolution and a regional implementation of HYCOM at 0.04° (available for the MHI only) resolution in addition to the MITgcm (S1 File).

### Statistical analysis

We are focusing on potential connectivity in this study, which estimates the connectivity of a site using physical oceanographic attributes and limited biological factors influencing dispersal ability (in our case PLD and habitat) [57,58]. To evaluate patterns of potential connectivity in the Hawaiian Archipelago, we created a connectivity matrix that measures the likelihood of particle exchange by currents among sites. The model generates a 687 x 687 settlement matrix (*Sij)* containing the number of particles released from habitat *i* (source site) that successfully reached habitat *j* (receiving site) for the full run of the model (five years). To create the rearward probability matrix, we scaled *Sij* to island specific total released particles. Rearward probability matrices report origin sites of particles arriving at the receiving site and can be written: P_ij (rearward)_ = S_ij_/∑S_j_.

We then binned the 687 habitat sites used in the dispersal model by island, resulting in a 31 x 31 island matrix, to allow for a simpler comparison of potential connectivity. The resulting probability matrix (*P*_*ij*_) shows the origin island of successfully transported particles at each island. The number in each cell of the *P*_*ij*_ matrix is the probability of a particle transported to island *j* having originated from island *i* for the five years the model was run, and each row in the matrix sums to 1. The diagonal of the probability matrix shows the self-recruitment for each island. Forward probability matrices were also generated and are described in S1 File. Because the majority of coral reef fish spawn during May-June [59,60], we calculated all metrics on both year-round releases and releases restricted to May-June of each year. All matrices were plotted using the software program Generic Mapping Tools (GMT) 4.5.11 [61].

Subtraction matrices were generated by subtracting the probability matrix for year round releases from the matrix for May-June releases using the subroutine *grdmath* in GMT 4.5.11. The resulting subtraction matrix shows where the two connectivity matrices differ. Only forward matrices were compared with each other, and rearward matrices with each other. We used mantel tests for each pair of connectivity matrices using function *mantel* in the *Vegan* package version 2.2–1 in the statistical software R [62] to calculate the correlation between the probability matrices.

Successful transport, defined as any particle within 5km of the center of a habitat pixel on day 45 after release, was calculated by tallying the daily number of successfully transported particles for all islands and dividing it by the total number of daily particles released for the five-year model run, allowing us to determine annual and seasonal variability. Additionally, we calculated island specific “settlement” success over five years.

Dispersal distance, the geographic distance between the release site and receiving site for a successfully transported particle at the receiving site, was determined by first calculating distances between all 687 habitat pixels using the distance matrix function *distm* with the *Haversine* formula in the R-package *geosphere* [63]. The *distm* function calculates the great circle distance (Haversine formula) between two points using their latitudes and longitudes in degrees and creates a 687 x 687 distance matrix (*Dij*) with the release sites (*i*) on the x-axis (rows) and receiving sites (*j*) on the y-axis (columns). We multiplied the settlement matrix (*Sij*) generated by the biophysical model (see above) with the distance matrix (*Dij*) to generate a product matrix (*Pij*). Because there is more than one spawning and settlement site (henceforth habitat site) per island (for example, Big Island has 129 habitat sites, Oʻahu has 62 and Kure Atoll has 13), we added all the distances for all the habitat sites in the product matrix belonging to each island, generating a 31x31 matrix containing the sum of all the distances of all the particles for each island called the island product matrix (*PIij)*. The same procedure was followed to generate an island settlement matrix (*SIij*); a square 31x31 matrix containing the total number of successful settlers for each island. We then divided the column sums from the island product matrix with the column sums of the island settlement matrix to obtain the mean dispersal distance for successfully settled particles at each island. These calculations were performed for year-round releases, as well as for particle releases confined to May and June of each year to allow us to explore seasonal patterns.

Self-recruitment, defined as the proportion of successfully transported particles at each island that originated from that same island, is an important metric when evaluating the persistence of a population [8,64]. We calculated self-recruitment for the duration of the model run for each island by dividing the number of released particles from an island that were transported back to that island by the total number of “settlers” there. This allows us to determine how dependent an island is on recruitment from outside locations to maintain the population.

Source-sink dynamics were assessed by calculating a source-sink index following Holstein et al. [21]. We define a source as an island that exports (outgoing) more particles than it imports (incoming), and a sink island imports more particles than it exports [21,64]. The source-sink index is a ratio of the difference between successful transport out of the island (export) and successful transport into the island (import), divided by the total of all successfully transported particles in and out of the island [21,64]. Because the index looks at the difference in the total flux of particles into and out of each island, it allows us to compare islands with varying amount of habitat and islands that have total numbers of transported particles that differs by orders of magnitude. The index spans from -1 to 1, and a positive index implies a source site and a negative index imply a sink site. The stronger the index the more likely the site is to be a persistent source or sink site. A zero index indicates that the flux of particles that are successfully transported onto the island and out of the islands are the same. This index allows us to compare islands in the archipelago and evaluates source-sink dynamics on a regional scale, whereas self-recruitment allows us to characterize islands as sources or sinks on a local scale.

## Results

### Potential connectivity

**Fig 2:**
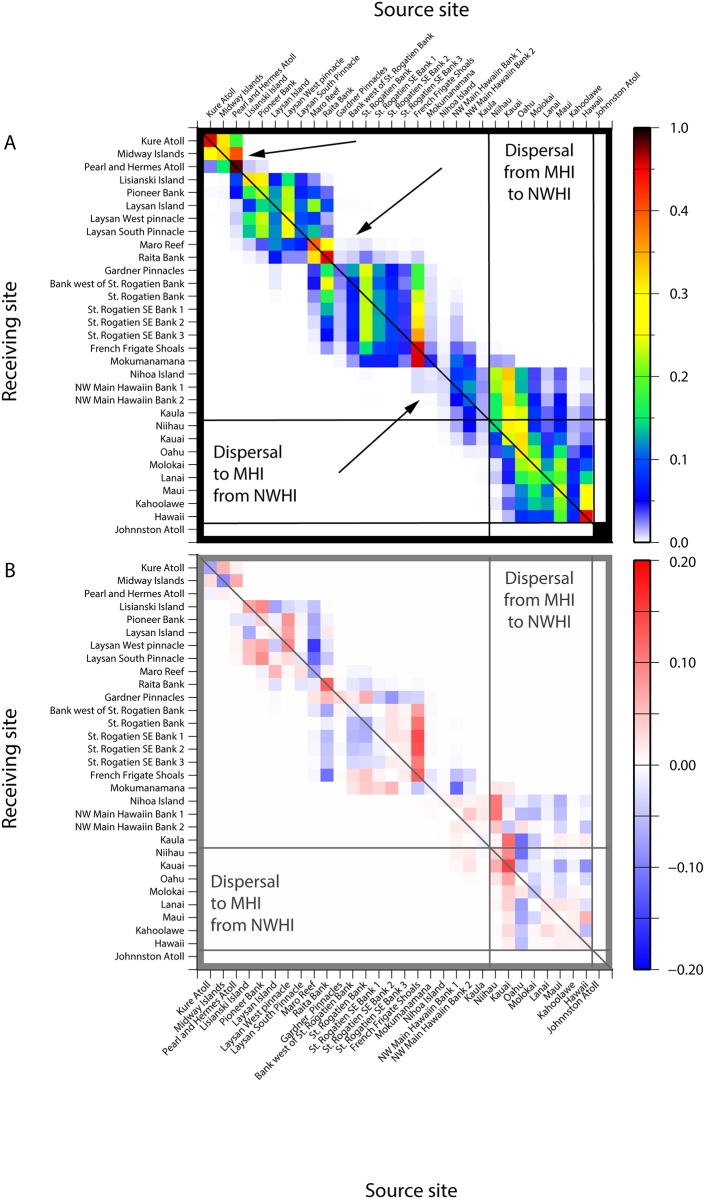
Potential Connectivity matrix for the Hawaiian Archipelago. (A) The values in each cell are “settlement” probabilities scaled to the receiving site for year-round particle release. Arrows indicated the breaks mentioned in the text. Each row in the matrix adds up to 1. High values (red) indicate high connectivity and low numbers (blue) indicate low connectivity, and white cells denote no connectivity. (B) Difference matrix showing the difference in connectivity between year-round and May-June particle release. The May-June release matrix is subtracted from the year-round release matrix (in A above). Positive values (red) denote a higher connectivity value for year-round releases and a negative number (blue) denotes higher connectivity for May-June released particles.

The probability matrix shows an isolation-by-distance pattern with sites far away from each other having little or no potential connectivity and considerable self-recruitment for most islands across the Archipelago (Fig 2). Restricting particle release to the typical May-June spawning season minimally alters the overall potential connectivity patterns (r = 0.932)(Fig 2b, S1 Fig). During spawning season O‘ahu and Maro Reef show stronger connections with neighboring islands while Ni‘ihau and Kauaʻi become less connected. Self-recruitment is more important for Kure and Midway Atolls (Fig 2) during spawning season, whereas Raita is more dependent on self-recruitment year round (Fig 2).

There is limited potential connectivity between the NWHI and the MHI, and the direction of dispersal is predominantly from the MHI to the NWHI. Particles originating in the MHI form 37 unique connections with sites in the NWHI (marked area in the upper right corner of Fig 2a), while particles originating from the NWHI only form 24 unique connections with sites in the MHI (marked areas in the bottom left in Fig 2a). Four times as many particles are successfully transported from the MHI to the NWHI than vice versa (3.1% from MHI to NWHI and 0.77% from NWHI to the MHI of the total successfully settled larvae). The MHI do not export any particles northwest of Mokumanamana, and islands located between Kaula and Nihoa in the center of the archipelago are the only islands in the NWHI to contribute particles to the MHI. Most particles released from Nihoa are lost to the system, indicated by the low self-recruitment (< 1%) and low contribution (6.067E-4%–0.72%) to the “settlement” at nearby islands (Fig 2).

While transport between the MHI and the NWHI is predominantly northwestward, total transport between all islands in the archipelago is reversed, with 37% of the successfully transported particles arrive at islands to the south (islands below the diagonal in Fig 2a), while 32% of particles are transported to islands to the north (islands above the diagonal in Fig 2a). However, 96.3% of the successfully transported particles originating in the MHI end up settling within the MHI and 3.73% are successfully transported to the NWHI, while 95.1% of successfully transported particles originating in the NWHI are successfully transported to sites within the NWHI and 4.87% of particles are successfully transported to the MHI.

There are three breaks in the connectivity matrix present for both year-round and seasonal particle release. Very few particles successfully cross these breaks. These breaks are more pronounced during spawning season releases (Fig 2b), and are more distinct in the forward matrices (S2 and S3 Figs). The southernmost break located between Nihoa and Mokumanamana is the most pronounced. No particles cross this break into or out of the MHI, effectively cutting the MHI off from the NWHI. The central break at Gardner Pinnacles and Maro Reef is traversed only by particles to and from Raita Bank. The northern break between Lisianski and Pearl and Hermes Atoll effectively isolates Kure Atoll and Midway Islands, resulting in high self-recruitment for the northernmost islands in the archipelago.

Using flow fields from different oceanographic circulation models at different spatial resolutions allows us to test whether the potential connectivity patterns are robust to model resolution. There is a strong correlation between the potential connectivity described above and the connectivity matrix generated from a dispersal model run that used current velocities from the coarser global HYCOM (r = 0.9291)(S4 Fig). For the MHI, we compared connectivity matrices generated from three dispersal model runs that used current velocities from the 0.04° MITgcm (S5a Fig), 0.04° regional HYCOM (S5b Fig), and 0.08° global HYCOM (S5c Fig). Potential connectivity for the MHI generated from the model run using different resolutions of the HYCOM currents showed the strongest correlation (r = 0.974)(S6a Fig), followed by connectivity matrices generated from the model runs with the same spatial resolution of the flow field, MITgcm and 0.04 regional HYCOM (r = 0.9533)(S6b Fig). We observed the largest difference between potential connectivity generated from model runs using MITgcm and the 0.08 km HYCOM flow fields (r = 0.9305)(S6c Fig).

### Total “settlement”

**Fig 3:**
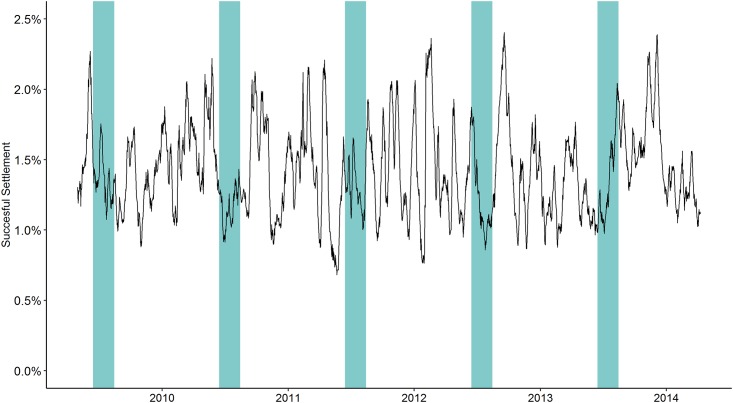
Total percent successful settlement for all sites in the Hawaiian Archipelago for the five-year model run. The green bars represent particles spawned during peak spawning season May-June each year.

Successful transport across all islands is highly variable with a mean of 1.416% (SE 7.708e-5) of all released particles successfully arriving at a receiving site over the five-year model run. The lowest total successful transport was seen on July 6, 2011 (0.682%), and the highest total successful transport on November 2, 2012 (2.405%). There is no discernible seasonal pattern in total arrivals observed for the archipelago as a whole (Fig 3). The highest rates of successful transport in 2009 (2.27%) and 2010 (2.22%) coincided with particles released during peak spawning season (marked by green bars in Fig 3); however, the following three years had some of the lowest rates of successful transport for particles released in May-June (0.68%, 0.95%, and 0.96%).

**Fig 4:**
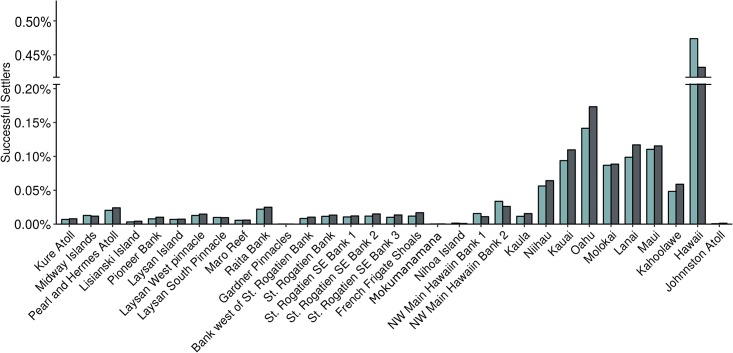
Total percent successful settlement at each island for the five-year model run. Green bars show settlement for particles spawned during May-June, gray bars show settlement for year-round spawning.

The MHI have overall larger relative probability of successful arrivals while islands near the observed breaks in the connectivity matrix have the lowest relative probability of successful arrivals. Johnston Atoll has the lowest relative successful arrival value of all at 0.0637% for year-round release and 0.0337% for peak spawning season releases (Fig 4). At Kaula, the relative arrival success is almost two orders of magnitude larger compared with Johnston Atoll, with 3.574% for year-round spawning. We see the largest relative arrivals for seasonal release at Lānaʻi with 2.713%. Hawaiʻi Island is the only MHI to show higher arrival success for particles released during spawning season (2.523%) compared to year-round releases (2.298%).

### Distance traveled

**Fig 5:**
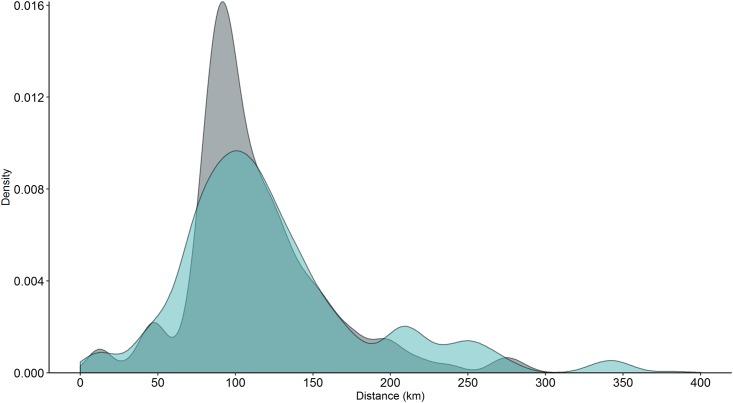
Density kernel for dispersal distance from source site for all islands for the five-year model run. Green kernel denotes May-June spawning and gray kernel year-round spawning.

The spatially averaged mean distance traveled is 112.32 km (SE = 1.705) for year-round particle release. Particles released during peak spawning season travel further, with mean distance of 124.37 km (SE = 2.372). Median distances are shorter, 101.39 km and 110.80 km for year-round and May-June release respectively, indicating that a few particles disperse, traveling significantly longer distances and driving up the mean. This is also evident from the long right tail on the density kernel (Fig 5).

**Fig 6:**
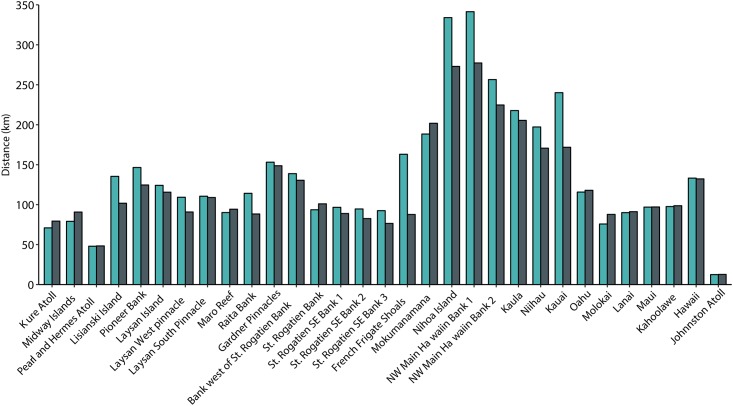
Island specific mean particle dispersal distances from the source island. Green color denotes particles released during May-June and gray denotes distances for year-round release.

Particles arriving at islands in the center of the archipelago travel the longest mean distances, while particles at Johnston Atoll travel the shortest (100% self-recruitment) (Fig 6). Particles successfully transported to the bank just south of Nihoa dispersed more than double the mean distance for other sites across the archipelago: on average 341.4 km during May-June release, and 277.2 km during year-round release. Consistent with total dispersal distances for all islands, island-specific dispersal distances are greater for particles released during spawning season, for 23 out of 31 islands (Fig 6). In the MHI, dispersal distances are consistent throughout the year except for particles released from Kauaʻi which has a much longer dispersal distance during May-June release. Kauaʻi dispersal distances are more similar to islands located in the center of the archipelago, likely due to the predominantly northwest direction of dispersal (Fig 2) and the longer distances between habitats in the Northwestern Hawaiian Islands. Particles released from island located to the northwest of each connectivity break (Pearl and Hermes Atoll, Maro Reef and Mokumanamana Island) have shorter dispersal distances compared to the island just southeast of the break (Lisianski Island, Gardner Pinnacles, and Nihoa Island) by 45.6%, 63.4%, and 73.9% respectively.

### Self-recruitment

**Fig 7:**
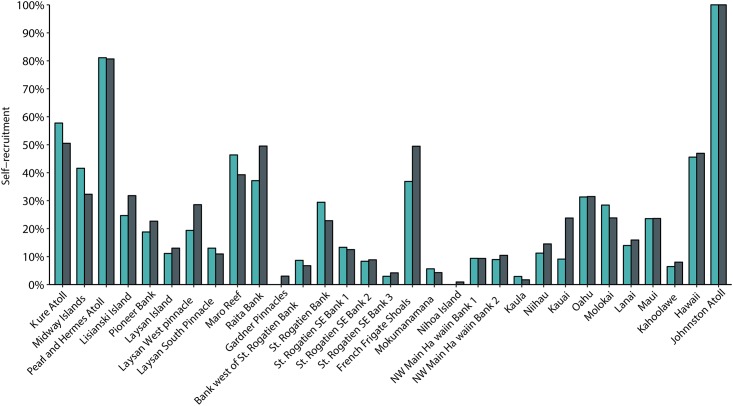
Island specific self-recruitment for the five-year model run. Green bars show self-recruitment for particles spawned during May-June, gray bars show self-recruitment for year-round spawning.

The mean self-recruitment for the archipelago is 25.2% (SE = 0.0414) but varies greatly from island to island. Johnston Atoll relies solely on self-recruitment (100%) for population persistence, while at Nihoa Island self-recruitment accounts for less than 1% of total settlement (Fig 7). During peak spawning season Nihoa, along with Gardner Pinnacles, import all their particles. Island specific self-recruitment (Fig 7, and diagonal in the connectivity matrix in Fig 2) is strongest at Kure (year-round 50.5%), Pearl and Hermes Atoll (year-round 80.67%, May-June 87.10%), Raita Bank (year-round 49.52%), Maro Reef (May-June 56.22%), French Frigate Shoals (year-round 49.46%, May-June 47.04%), Hawaiʻi Island (year-round 46.93%, May-June 42.44%) and Johnston Atoll (year-round and May-June 100%). These high self-recruitment islands are located either to the north of connectivity breaks or at the edges of the archipelago.

### Source-Sink dynamics

**Fig 8:**
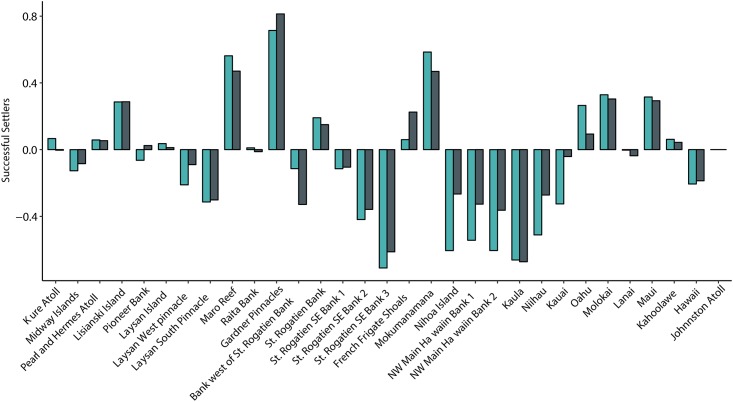
Source-Sink index for all islands in the Hawaiian Archipelago for the 5-year model run. Positive numbers indicate a net source location, and negative numbers indicate a sink location. Green color denotes particles released during May-June of each year and gray denotes distances for year-round releases.

The Source-Sink Index weighs the successful “settlers” originating from an island against the successful “settlers” coming to that same island, and gives a good indication on what role an island plays within the archipelago. A positive index indicates that a site exports more particles than it imports, and is thus considered an important source site. Conversely, a negative index means a site imports more particles than it exports, and should be classified as a sink. For year-round releases, 16 islands had a negative index and 13 islands had positive indexes; Kure and Johnston Atolls each had an index of zero because virtually all settlers to these sites are a result of self-recruitment (Fig 8). Gardner Pinnacles had the strongest positive index, followed by Maro Reef and Mokumanamana island, indicating that they are persistent source sites. The middle of the archipelago, from Niʻihau to St. Rogatien, are predominantly sink islands, with Kaula having the strongest negative index. Because the source-sink index is a ratio between particle flux into and out of an island, an island with low self-recruitment can still have a positive index (net source) if it exports more successful particles than it imports. All islands but three kept their source or sink assignment when comparing year-round releases to May-June releases. Pioneer Bank and Laysan Island act as weak source sites for year-round releases, but for summer releases they act as sink sites. The bank west of St. Rogatien Bank is a sink during year-round releases but a source of particles during summer.

## Discussion

Our passive particle dispersal model identified three predicted connectivity breaks in the Hawaiian Archipelago, and these regions of limited connectivity coincide with observed patterns of genetic structure reported for coral reef species in the Northwestern Hawaiian Islands (NWHI). But, multiple genetic breaks observed in the inhabited MHI are not explained by passive dispersal, rather our model predicts that the MHI should be well-mixed. It is always desirable to parameterize a model with as much accurate biological data as possible [65], but in the absence of reliable data, a simple physics driven model can provide important information on the interaction of particles with the physical environment [66–69]. While obviously desirable for future studies, we have not incorporated any ontogeny, behavior or mortality into the model because such data are scarce for local fish and invertebrate species. Wren & Kobayashi [42] ground-truthed the dispersal model using trawl surveys off Big Island, which showed that a simple physics driven dispersal model is able to predict observed larval fish distributions for the region. The predominant effect of incorporating realistic larval behaviors into oceanographic models to date is reduced passive dispersal and enhanced self-seeding [70– 75]. Even without larval behavior, our results show a surprising predominance of self-recruitment for the Hawaiian Islands, with more limited exchange than by conventional wisdom, so we predict that explicit incorporation of larval behaviors in future iterations of the model will only enhance that trend for islands throughout the Hawaiian Archipelago.

### Potential connectivity

Isolation by distance (IBD), where genetic differentiation increases with increasing geographic distance [76], is often considered the norm in marine population genetics, especially for linear coastlines or chains of islands [77–79]. The Hawaiian Archipelago, a linear string of islands far removed from outside genetic influences, is the ideal place to study IBD due to its stepping stone configuration [80]. As expected, we observed an IBD pattern of particle exchange in this study, indicated by the decrease in potential connectivity with increasing distance (Fig 2). There is also a striking degree of self-recruitment driven entirely by physics of passive particles in this system, with the vast majority of potential connectivity in the matrix falling along the diagonal. However, researchers focusing on genetic studies have failed to recover an IBD pattern from *F*_*ST*_ in a majority of species; rather a regional pattern of differentiation between the MHI and NWHI is more evident [22]. Given the differing scales of the analyses, the primary breaks in the potential connectivity model correspond well to breaks in genetic structure, particularly the split by Mokumanamana-Nihoa and the far northwestern islands of the Archipelago [20]. There was no indication of obstacles to the exchange of particles among islands within the MHI, although genetic approaches reveal consistent barriers to exchange among neighboring islands [20]. This may indicate that physical oceanographic drivers of dispersal are trumped by other, most likely biological, drivers [81], or that more sensitive techniques are required to recover a significant IBD signal from data with regional structure (e.g., [82]). The congruence between our modeling results and genetic analysis in the NWHI, but not in the MHI, begs the question of what ultimately controls connectivity in this system. Does the lack of congruence in the MHI have to do with the imprecision of the oceanographic model, the lack of biological realism and larval behavior in the model, the differences between the high main Hawaiian Islands and the low-lying atolls of the NWHI, or the strong ecological differences and anthropogenic impacts that differentiate the MHI and NWHI? These questions provide fertile ground for future studies to determine statistical drivers of observed breaks in areas that appear to be within reach for routine larval dispersal [81].

The current management strategy for the MHI is based, at least in part, on the unfished stock in Papahānaumokuākea Marine National Monument that is expected to spill over and replenish fished stocks in the MHI. Our results, coupled with previous genetic work, do not support this expectation, warranting revision of stock boundaries and resource management plans. A growing number of studies support directional dispersal in the Hawaiian Islands for corals [15,36,83], limpets [37], cucumbers [38], and fish [35]. These studies use Eulerian and Lagrangian dispersal modeling, population genetic techniques, or both. Congruence between different studies and study methods lends credence to the emerging idea that dispersal in the Hawaiian Archipelago is primarily directional, from the MHI to the NWHI. While the number of unique connections from the MHI to the NWHI were 65% higher than from the NWHI to the MHI, and 60% of the habitat is located in the MHI, the shallow reef fish biomass in the NWHI is 260% greater than in the MHI [84]. Although the probability of larval transport is greater from the MHI to the NWHI and not the other way around, it is important to note that the total number of larvae transported may in fact be greater from the NWHI down to the MHI when accounting for the larger standing stock biomass in the NWHI.

The predominant surface currents (Fig 1) in the Hawaiian Archipelago, the Hawaiʻi Lee Current (HLC) and the North Hawaiian Ridge Current (NHRC), flow along the flanks of the MHI then continue westward. A possible barrier preventing transport between the MHI and the NWHI is the NHRC/HLC Extension that parts from the Hawaiian Islands just north of Kauaʻi, near 22°N, diverting waters west across the pacific [85]. The location of this current extension coincides with the location of the potential connectivity break suggested here between the MHI and the NWHI, between Mokumanamana and Nihoa.

**Fig 9:**
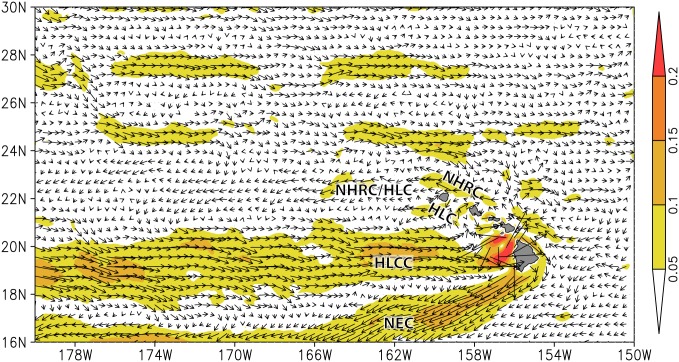
Modeled flow fields produced by the regional Hawaiʻi MITgcm for May 2009-May 2014. Surface geostrophic currents (m/s) around the Hawaiian Archipelago. The eastward zonal flows are visible near 25°N and 27°N. Major surface currents are marked: Hawai‘i Lee Counter Current (HLCC), Hawai‘i Lee Current (HLC), North Equatorial Current (NEC) and North Hawaiian Ridge Current (NHRC). Vectors show current velocities and colors denote current speeds.

The presence of zonal flows in the Pacific [86] might influence transport and potential connectivity patterns. The regional implementation of the MITgcm shows two locations with zonal flows near 25°N and 27°N (Fig 9). Water is moving eastward in these areas and may pose a barrier to particle transport. The connectivity breaks in the NWHI are located between Raita and Gardner near 25°N, and between Lisianski and Pearl and Hermes near 27°N. The zonal flows seen in the MITgcm flow field are not present in the global HYCOM flow fields (S8 Fig) but the breaks are still present in the probability matrix from the model run using global HYCOM currents (S4 Fig), indicating the zonal flows are not solely responsible for the connectivity breaks. The persistence in break locations may be due to the lack of suitable habitat around Mokumanamana and Nihoa, or an increase in mean distance between available habitat in these areas that further limit successful settlement.

### Total “settlement”

Mesoscale eddies that form in the lee of the Big Island of Hawaiʻi during summer have long been hypothesized to retain larvae near the Big Island, thus increasing the regional settlement probability for those larvae [44,59,87–89]. If this hypothesis were correct, we would expect to see increased settlement success and higher self-recruitment for the Big Island during particle releases in summer months when mesoscale eddies are common. Our results do not support this hypothesis however, because we see the opposite effect: self-recruitment for BI was lower for particles released during May-June, indicating that the eddies are not retaining particles released from the Big Island and transporting them back to the Big Island. Our findings are congruent with recent studies conducted by Fox et al. [90] and Vaz et al. [43] who likewise found no relationship between eddy activity and recruitment on the Big Island, and together these studies call into question the longstanding hypothesis that eddies increase regional settlement on the Big Island. Both Fox et al. and Vaz et al. focused solely on the Big Island and did not investigate recruitment or connectivity between BI and other islands. In direct opposition to the hypothesis, our model shows that self-recruitment was not enhanced but rather that successful transport to other islands was higher for particles released from the Big Island during May-June. This stands in stark contrast to other islands throughout the archipelago where total successful transport is higher for particles released year-round. We propose an alternative hypothesis: the eddies do not appear to increase retention and self-recruitment of larvae on the Big Island, but instead may facilitate transport and increase settlement of BI larvae on other islands in the archipelago by capturing particles released on Big Island and moving them up the island chain.

### Distance traveled

The particles that dispersed the farthest in our study traveled in excess of 250 km (equivalent to the distance between Kona on the Big Island and Honolulu on O‘ahu), and originate on Mokumanamana, Nihoa and the NW Main Hawaiian Banks. These long-distance dispersers influence gene flow and can prevent genetic differentiation in a population [91], meaning that the long tail distances can potentially be more informative than mean distances in the context of population resilience. Seasonality analysis showed that during summer releases this distance increased by one third, to more than 350 km. Both mean and maximum dispersal distances increase for particles released during the summer, in part, because self-recruitment is lower at this time, so the mean distance traveled by successful settlers tends to be longer. This seasonal difference has implications for population persistence, because self-recruitment allows for local adaptation but leaves a population more vulnerable to local disturbances. Summer months may play a disproportionately important role in long-range dispersal, and the majority of spawning in coral reef fish populations take place during these months. There is less information about spawning seasons of invertebrates, but coral spawning is clearly more variable, with some species spawning year-round and other species having peak release between the months of April through September [92–96]. The inclusion of realistic larval behavior in the model is expected to shorten mean dispersal distances given the wealth of studies showing that larvae tend to minimize passive dispersal and orient and swim towards settlement habitat (reviewed by [65,70,97–102]). Therefore, potential dispersal distances reported here are likely over-estimates of realized dispersal distances achieved by larvae in the Hawaiian Archipelago. The greatest management unit scale for the archipelago should be more conservative than the distances we report, and limited to less than 150 km, the mean dispersal distance, because connectivity at greater distances is not only highly limited but typically driven by few individuals transported disproportionately long distances. Individuals in the long tail of a dispersal kernel may influence gene flow but are not expected to contribute anything to the persistence or demography of populations for management [5,8]. One such example is the connection between Johnston Atoll and the central Hawaiian Archipelago. Although exchange with Johnston Atoll is exceedingly rare, it does happen, and a few particles traveling the 1300 km between the Hawaiian Archipelago and Johnston Atoll drastically increase the average dispersal distance calculated from these simulations. The connection with Johnston Atoll is important as it provides a stepping stone ‘gateway’ into and out of Hawaiʻi for marine organisms [38,41,64,103], but does not represent an ecologically relevant source of propagules and should be excluded when evaluating the scale of resource management units for the Hawaiian Archipelago. Further, given that Johnston is the closest external reef habitat, it seems unlikely that more distant sites are better connected to the Hawaiian Archipelago, although some evidence points to similar rare long-distance dispersal from the northern Line Islands [104].

### Self-recruitment and source-sink dynamics

Pearl and Hermes, Kure Atoll, French Frigate Shoals, Hawaiʻi Island, and Johnston Atoll all have high self-recruitment (i.e., more than 40% of particles that “settle” at each of those island were released from that same island). High self-recruitment suggests that they can persist without propagule input from other islands and implies that these islands are less sensitive to regional disturbances. Conversely, these sites are at greater risk from local disturbances, and if local extinction occurs, they are not able to recover without external sources of larvae. At the other end of the spectrum, Gardner Pinnacles, Mokumanamana, and Nihoa Islands all have very low self-recruitment and low recruitment overall, making them much more sensitive to fluctuations in population size and larval supply. The high genetic differentiation of these sites relative to others [81] combined with this relatively small population size and low self-recruitment creates a high risk of local extinction and long-term population persistence appears to be primarily reliant on outside sources of recruits.

Self-recruitment describes population dynamics on each island, but to get a better idea of population dynamics on a regional scale and make informed management decisions, the source-sink index can be equally informative [21]. In the simplest terms, source sites are net exporters of larvae whereas sinks are net importers. A sink site needs nearby areas to provide propagules in order to persist, and thus management strategies for source and sink islands will be very different. For example, an island with a lot of available habitat, such as Hawaiʻi Island which accounts for roughly 1/6^th^ of all available coral reef habitat in the archipelago [52–54], will contribute many particles, but also has many more receiving habitats than a smaller area; thus, the Big Island can import a very large number of particles, and actually exceed its output. The source-sink index is useful when comparing islands with varying amounts of habitat because the ratio looks at both import into, and export out of, an island. It is also important to note that self-recruitment and the source-sink index are not mutually exclusive; an island with high self-recruitment can still serve as a source site for nearby islands, as is the case with Kure Atoll, Maro Reef, and French Frigate Shoals. In contrast, the Big Island, Laysan Pinnacles, and Midway Atoll have high self-recruitment but are also sink sites. These latter three islands warrant special consideration to maintain the high level of self-recruitment while also relying on the protection of nearby islands because recruitment subsidy is still important for population persistence.

### Connectivity breaks

We should take caution against broadly applying these connectivity findings to all species, because larval biology and ecology vary greatly for marine animals and those differences are known to influence ocean transport (reviewed by [65,70,97–102]). For example, our model results show that Nihoa Island depends strongly on importation of larvae to persist, however, among intertidal limpets (Cellana sp.), an invertebrate with a negatively buoyant and shorter lived veliger larva, estimates of self-recruitment are far higher (>90%) (Bird, pers. comm. 2016). In our efforts to examine the potential connectivity patterns in the Hawaiian Archipelago, we parameterized our model after a generic broadcast spawning reef fish with an “average” PLD of 45 days. It is important to recall that this generic fish is not representative of everything on the reef, and population genetic studies show some dramatic differences among species studied to date [20]. Here, we report potential population connectivity, and there are many biotic and abiotic factors influencing realized population connectivity (mortality, time to competency, metamorphosis, settlement, recruitment etc.) that may cause a mismatch between realized and potential connectivity [58,105,106].

In the Hawaiian Archipelago we can identify three breaks in potential connectivity throughout the archipelago: a southern break by Nihoa and Mokumanamana, a central break between Raita and Gardner Pinnacles, and a northern break between Lisianski and Pearl and Hermes Atoll. The NWHI breaks generated by our passive particle transport model are surprisingly consistent with population genetic breaks observed for fish and invertebrate species [20,107,108]. Interestingly, the major differences between the modeled potential connectivity and genetics are seen in the inhabited MHI, where modeled potential connectivity is not able to resolve observed genetic breaks.

There are many differences between the uninhabited low atolls and reefs of NWHI relative to the highly populated high islands of the MHI. However, one major difference between the MHI and NWHI that may impact our model results is the amount and quality of available habitat. The banks, pinnacles, and atolls in the NWHI are small, the largest being Midway Atoll at 6.2 km^2^, and north of Gardner Pinnacles, all are sandy and low lying. Close to 60% of the total coral reef habitat used in this study is located in the MHI (402 out of 687 pixels). In addition, the MHI have a more complex geology, large channels with strong currents, like the ʻAlenuihāhā channel that passes between Big Island and Maui, and mountain-wind interactions that change surface circulation, features mostly lacking in the NWHI. The regional implementation of MITgcm at 0.04° resolution does not resolve nearshore flow. It produces the typical subtropical gyre circulation in the NWHI, with zonal jets associated with propagating mesoscale eddies [86], whereas in the MHI, interactions between the high mountains and the northeasterly trade winds generate strong eddies [109] and a highly variable flow field overall (Fig 9). Recent studies have shown that mesoscale circulation features like eddies can create physical barriers to dispersal [26], and although MITgcm is eddy-resolving, there may be oceanographic features, especially in the MHI, causing genetic breaks in the MHI that are not well resolved in the circulation model. In addition to physical and oceanographic differences, behavior, selection, ecological and anthropogenic differences (e.g. [110,111]) among the MHI may explain the mismatch between the realized (inferred from genetic data here) and potential (modeled) connectivity, but are not necessary to account for the NWHI breaks. It is likely that there is a physical barrier to dispersal in the areas where population genetics and our dispersal modeling show congruence, whether it be channels [26], lack of suitable habitat or oceanographic features (reviewed by [112]). Expanding the biophysical model parameterization to include life history parameters, a settlement window instead of a fixed PLD, and realistic larval behavior are expected to enhance self-recruitment and may resolve some of the genetic breaks observed in the MHI not caused by seascape features.

## Supporting Information

S1 Fig Difference matrices comparing (A) forward and (B) rearward “settlement” probabilities between year round particle releases and releases during May—June only. Red indicated year round probabilities were higher and blue colors indicate releases during May -June only had higher probability of transport. White indicate no probability of transport.
(TIF)

S2 Fig Forward probability matrix for the model run using MITgcm currents. Colored tiles represent probability of transport from source sites to receiving sites. White areas indicate no probability of transport between source and receiving sites.
(TIF)

S3 Fig Potential connectivity matrices for particle tracking model run using 0.08° HYCOM currents for (A) forward probabilities and (B) rearward probabilities. Colored tiles represent probability of transport from source sites to receiving sites, scaled after receiving site with each row adding up to zero. White represents a zero probability of connectivity.
(TIF)

S4 Fig Difference matrices comparing (A) forward and (B) rearward transport probabilities between year round releases in the dispersal model run using 0.08° HYCOM and regional (0.04°) MITgcm. Red indicated HYCOM driven probabilities were higher and blue colors indicate the MITgcm driven model run had higher probability of transport. White indicate no probability of transport.
(TIF)

S5 Fig Probability matrices for forward (A1, B1, C1) and rearward (A2. B2, C2) potential connectivity for the Main Hawaiian Islands for three transport model runs. (A) is a subset of Fig 2a for the MHI, (B) shows probabilities from a model run using regional 0.04° HYCOM currents, and (C) is a MHI subset of S4 Fig. Colored tiles represent probability of transport from source sites to receiving sites. Forward matrices are scaled after receiving site with each row adding up to zero. White represents a zero probability of connectivity.
(TIF)

S6 Fig Difference matrices for the Main Hawaiian Islands. Matrices show for forward (A1, B1, C1) and rearward (A2, B2, C2) transport probabilities for year round releases in the dispersal model run between the regional MITgcm and 0.04 HYCOM (A), between 0.08° HYCOM and regional (0.04°) MITgcm (B) and between the two resolutions of HYCOM (C). Red colors indicated 0.08° HYCOM driven probabilities were higher in (B) and (C) and MITgcm in (A). Blue colors indicate the MITgcm driven model run had higher probability of transport in (B) and 0.04 HYCOM in (A) and (C). White represents no probability of transport.
(TIF)

S7 Fig Map showing averaged surface circulation from global 0.08 HYCOM generated data for the Hawaiian Archipelago. Major surface currents (m/s) are marked. Zonal flows in the NWHI are not present in this dataset.
(TIF)

S8 Fig Map showing averaged surface circulation from the regional 0.04 HYCOM generated data for the Main Hawaiian Islands. Major surface currents (m/s) are marked.
(TIF)

S1 File Supplemental methods and results for dispersal modeling in the Hawaiian Archipelago. This file contains supplemental methods and results for additional model runs and connectivity calculations.
(DOCX)
