# Supplementary figures and images for: Modeled Population Connectivity across the Hawaiian Archipelago
Source: PLoS One. 2016 Dec 8;11(12):e0167626. doi: 10.1371/journal.pone.0167626 (PMC5145177; doi:10.1371/journal.pone.0167626)

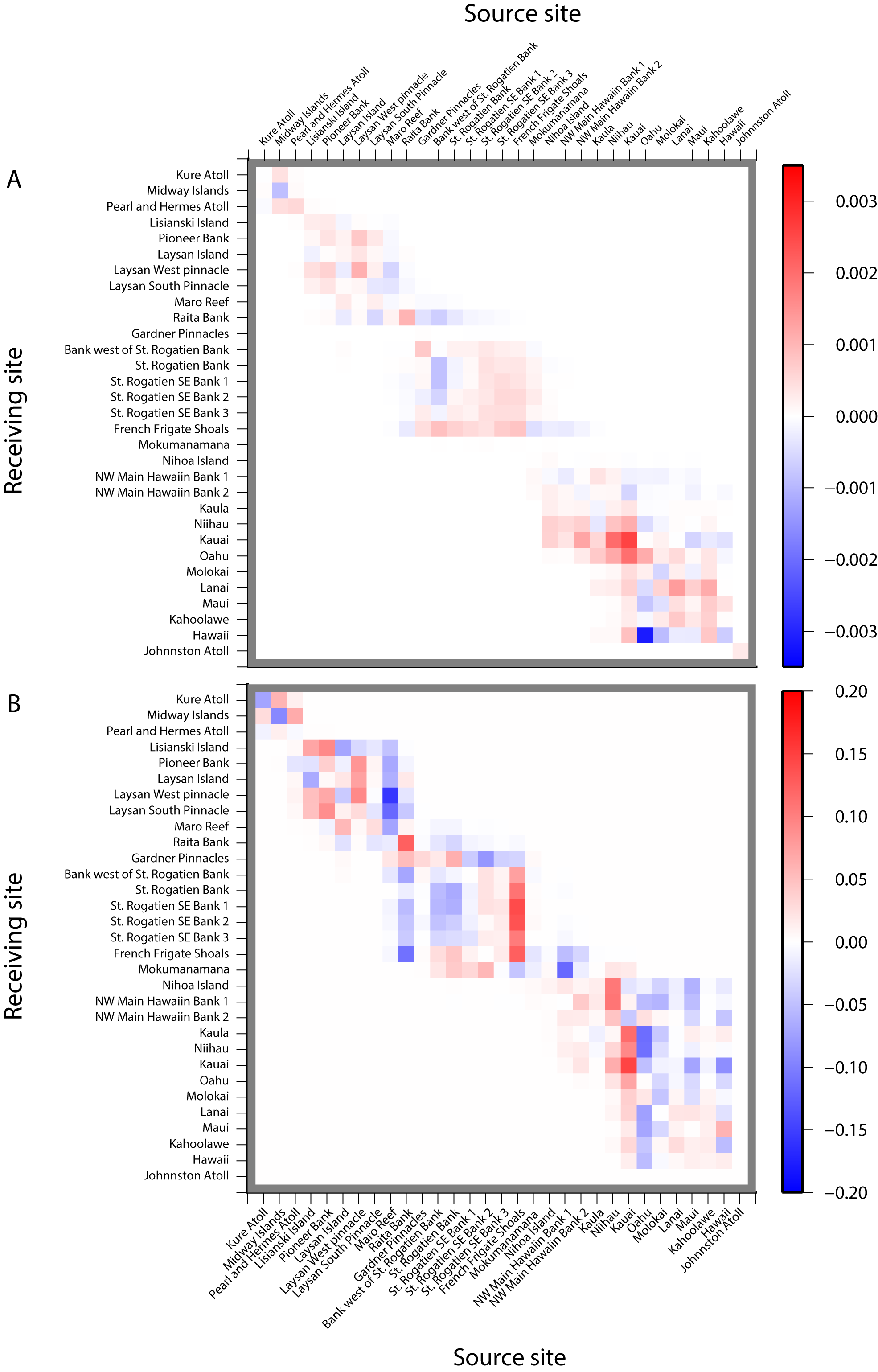

Supplement: S1 Fig — Red indicated year round probabilities were higher and blue colors indicate releases during May -June only had higher probability of transport. White indicate no probability of transport. (TIF) [file pone.0167626.s001.tif]

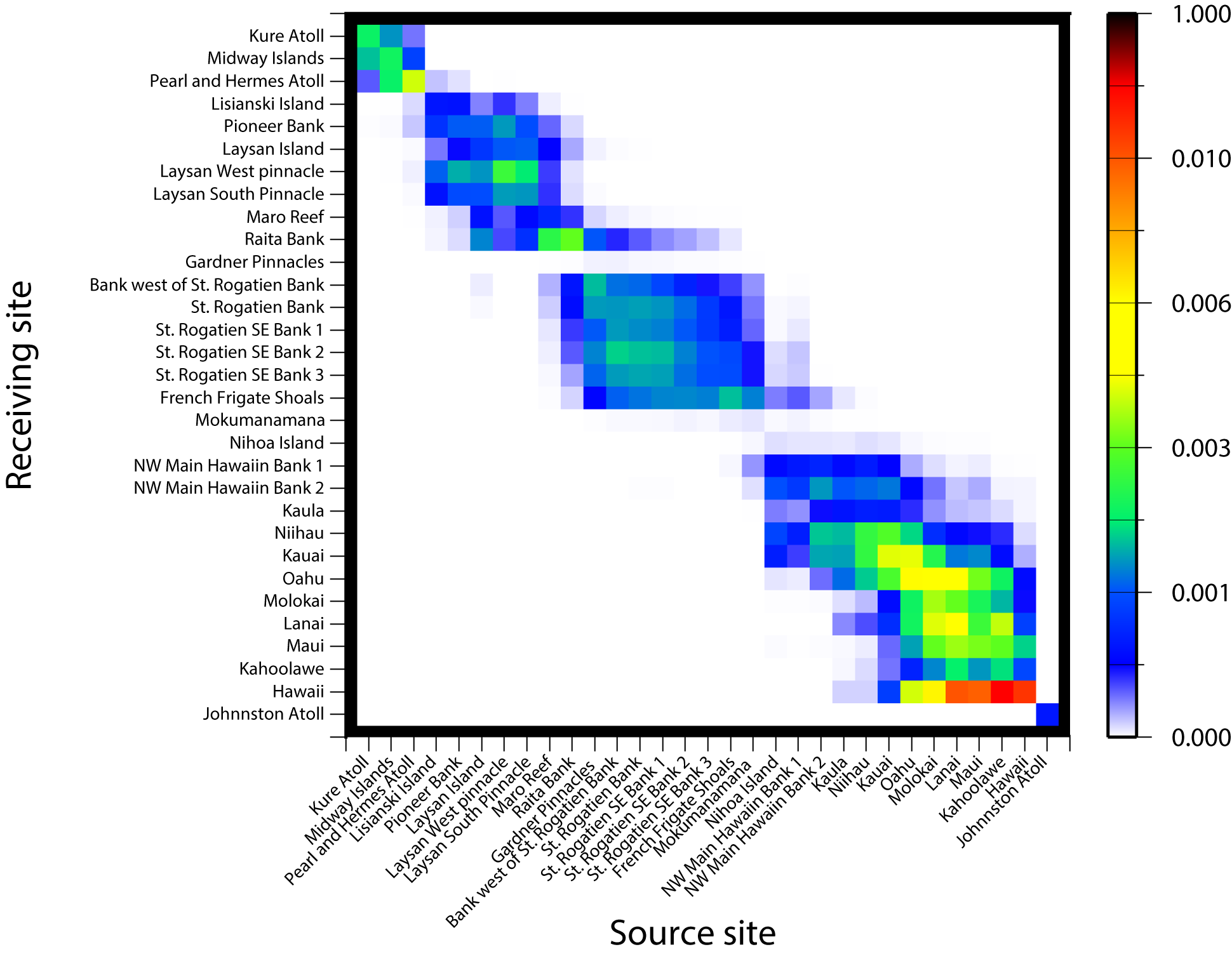

Supplement: S2 Fig — Colored tiles represent probability of transport from source sites to receiving sites. White areas indicate no probability of transport between source and receiving sites. (TIF) [file pone.0167626.s002.tif]

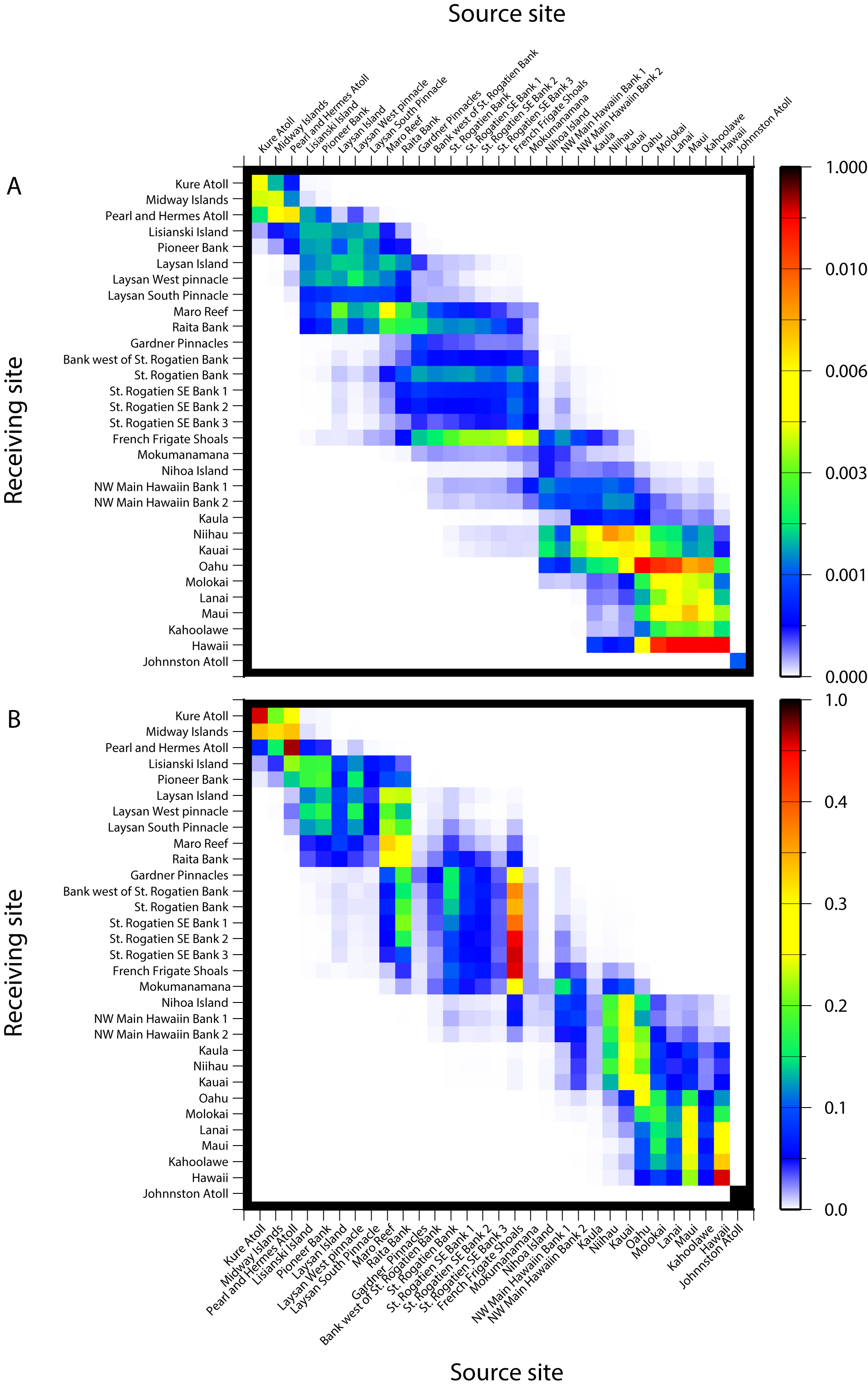

Supplement: S3 Fig — Colored tiles represent probability of transport from source sites to receiving sites, scaled after receiving site with each row adding up to zero. White represents a zero probability of connectivity. (TIF) [file pone.0167626.s003.tif]

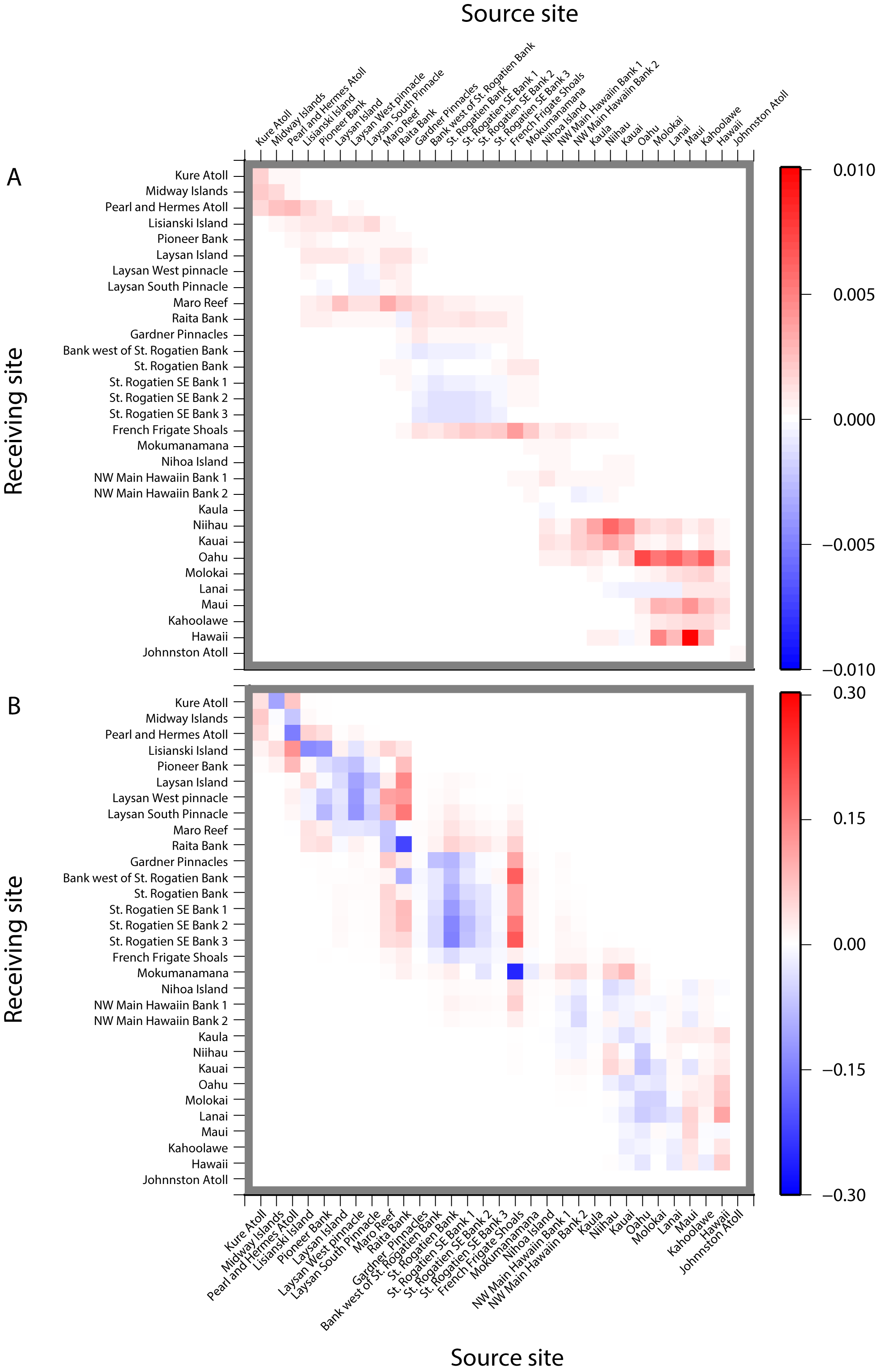

Supplement: S4 Fig — Red indicated HYCOM driven probabilities were higher and blue colors indicate the MITgcm driven model run had higher probability of transport. White indicate no probability of transport. (TIF) [file pone.0167626.s004.tif]

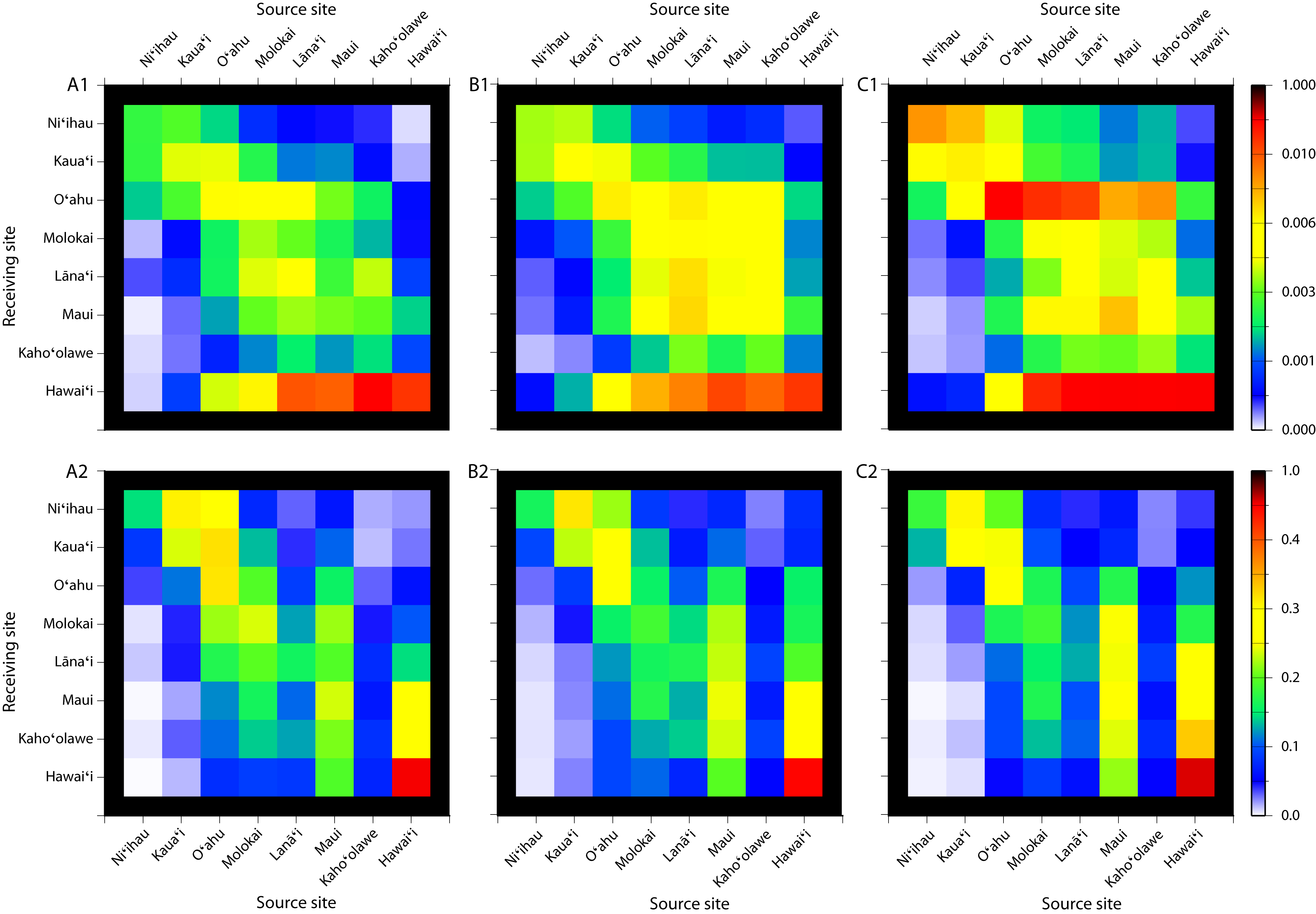

Supplement: S5 Fig — (A) is a subset of Fig 2a for the MHI, (B) shows probabilities from a model run using regional 0.04° HYCOM currents, and (C) is a MHI subset of S4 Fig. Colored tiles represent probability of transport from source sites to receiving sites. Forward matrices are scaled after receiving site with each row adding up to zero. White represents a zero probability of connectivity. (TIF) [file pone.0167626.s005.tif]

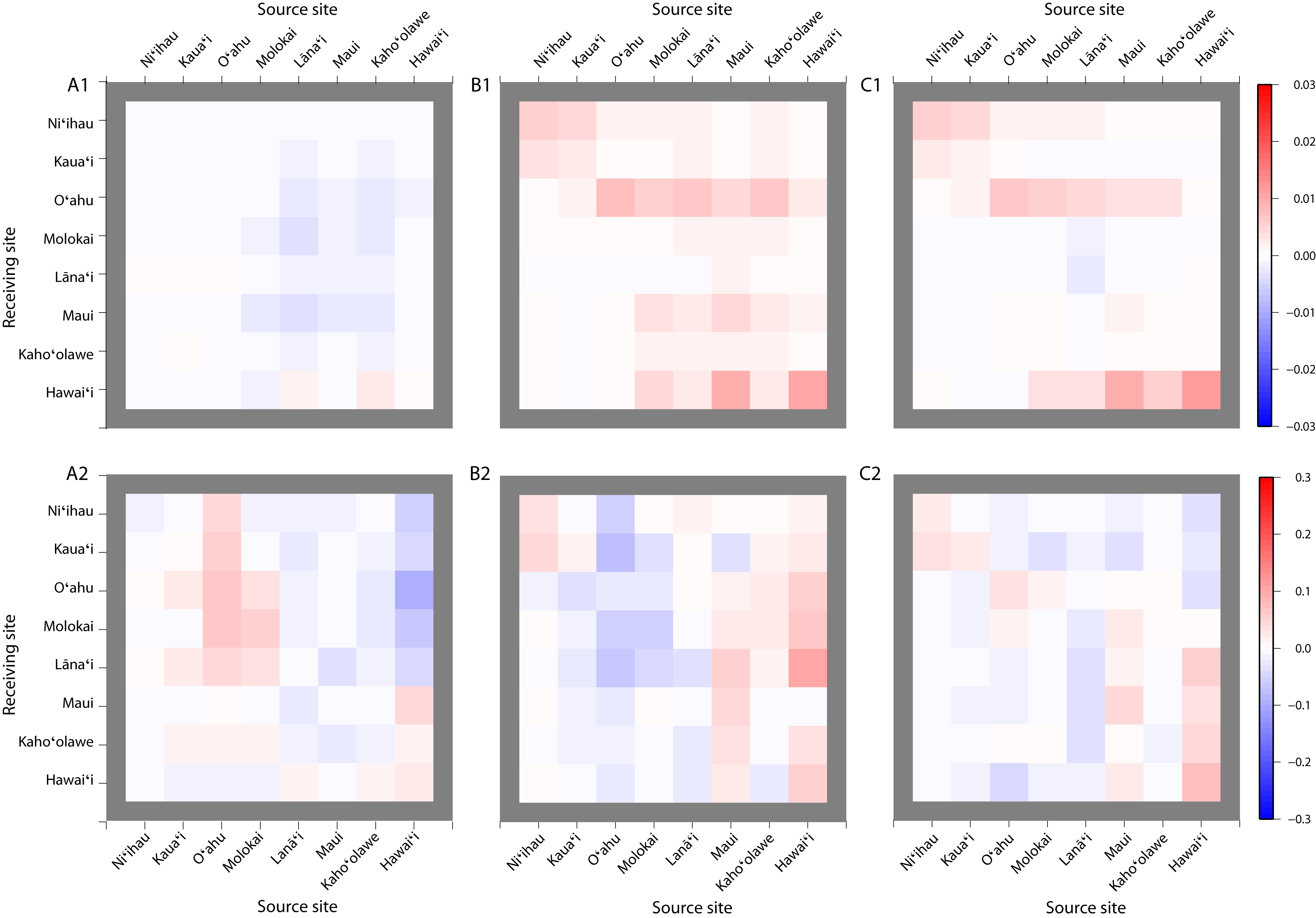

Supplement: S6 Fig — Matrices show for forward (A1, B1, C1) and rearward (A2, B2, C2) transport probabilities for year round releases in the dispersal model run between the regional MITgcm and 0.04 HYCOM (A), between 0.08° HYCOM and regional (0.04°) MITgcm (B) and between the two resolutions of HYCOM (C). Red colors indicated 0.08° HYCOM driven probabilities were higher in (B) and (C) and MITgcm in (A). Blue colors indicate the MITgcm driven model run had higher probability of transport in (B) and 0.04 HYCOM in (A) and (C). White represents no probability of transport. (TIF) [file pone.0167626.s006.tif]

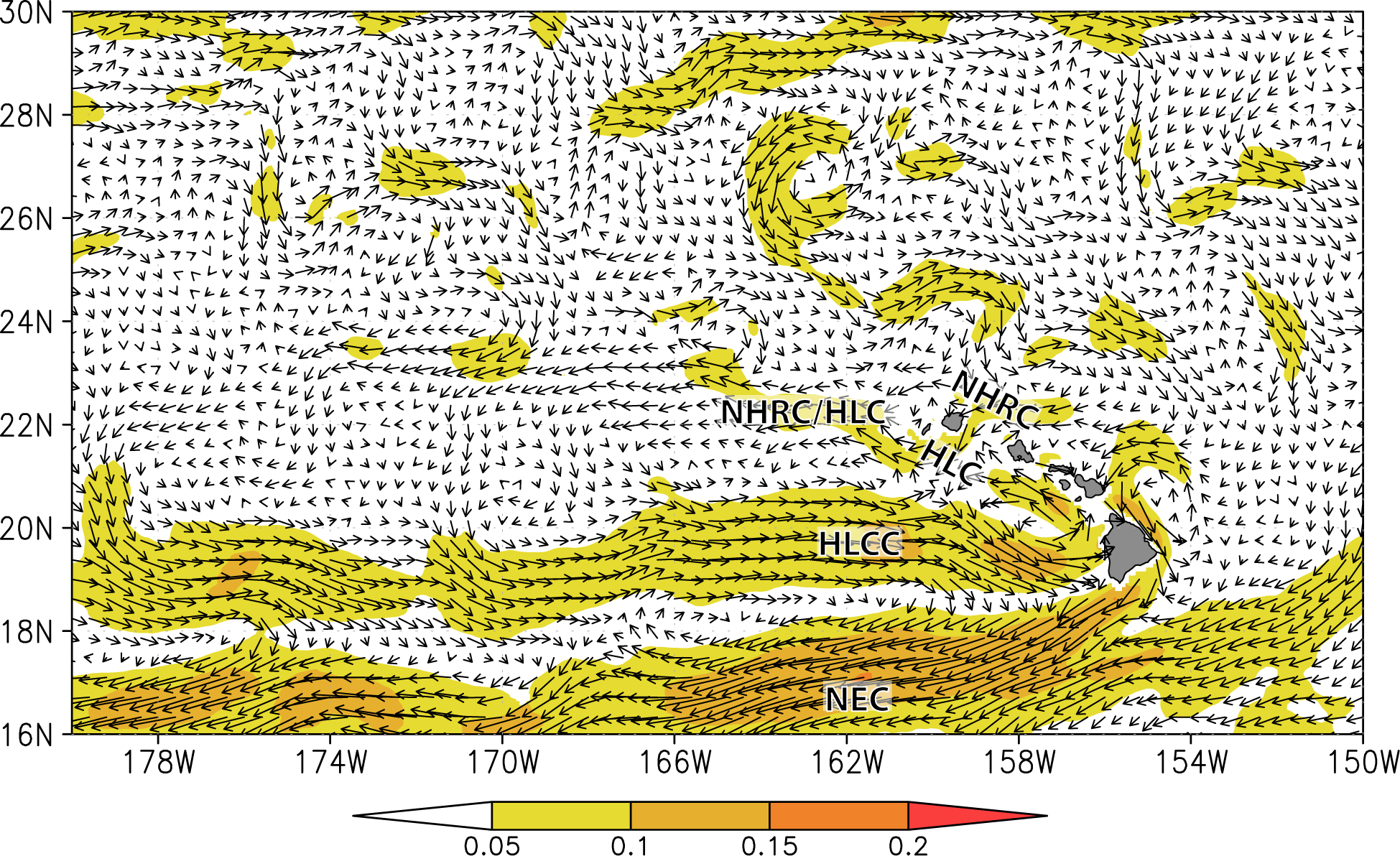

Supplement: S7 Fig — Major surface currents (m/s) are marked. Zonal flows in the NWHI are not present in this dataset. (TIF) [file pone.0167626.s007.tif]

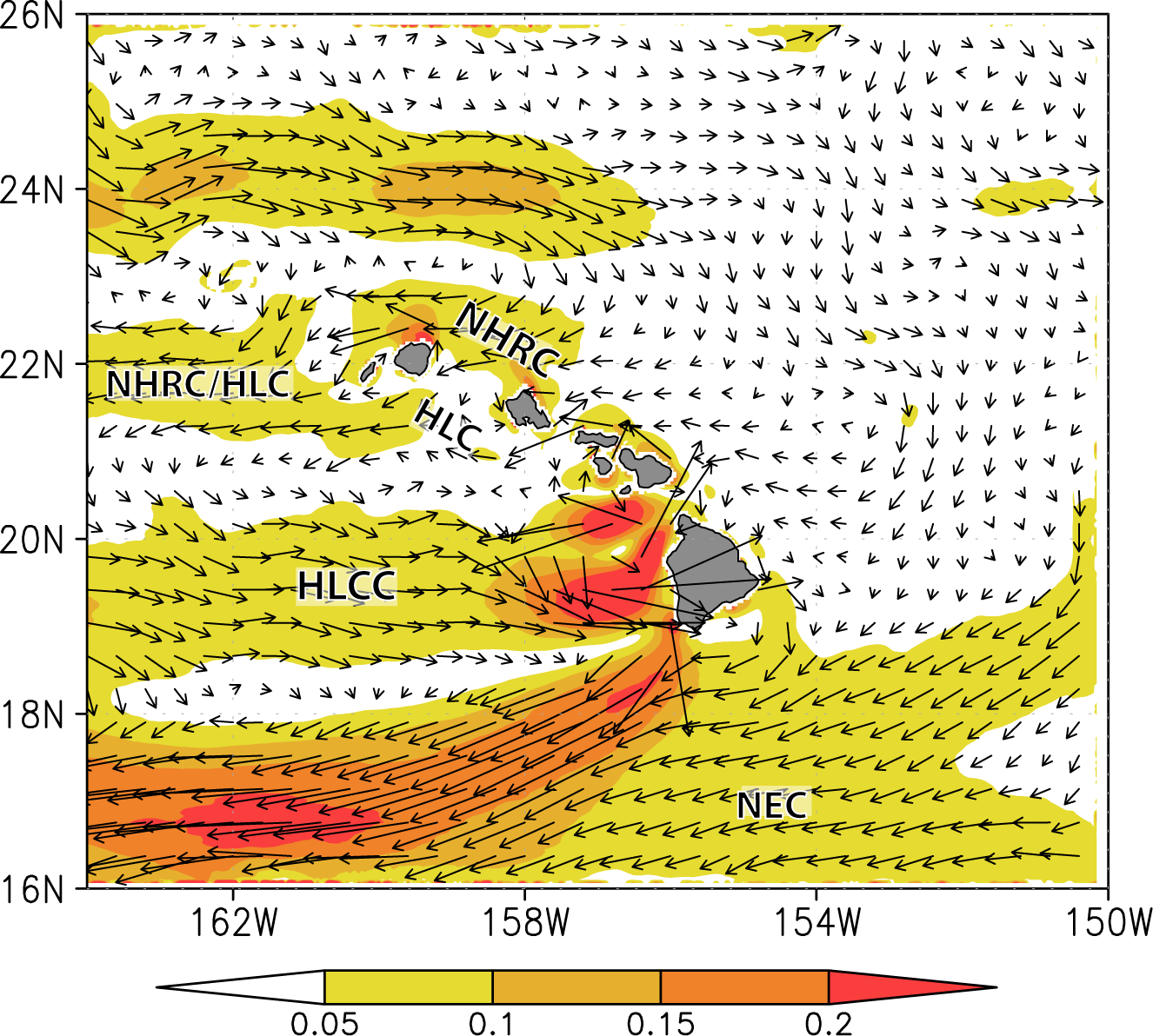

Supplement: S8 Fig — Major surface currents (m/s) are marked. (TIF) [file pone.0167626.s008.tif]
