# Supplementary material for: Modeled Population Connectivity across the Hawaiian Archipelago
Source: PLoS One. 2016 Dec 8;11(12):e0167626. doi: 10.1371/journal.pone.0167626 (PMC5145177; doi:10.1371/journal.pone.0167626)
Supplement: S1 File — This file contains supplemental methods and results for additional model runs and connectivity calculations. (DOCX) [file pone.0167626.s009.docx]

*Supplemental material for Modeled population connectivity across the Hawaiian Archipelago.*

**Methods**

*HYCOM*

The HYbrid Coordinate Ocean Model (HYCOM) is a global primitive equation ocean circulation model run by the HYCOM consortium [(Bleck 2002; Chassignet et al. 2009)](https://paperpile.com/c/xE5HWy/ftvD+JYyD). The Hybrid coordinate refers to the model's vertical coordinate system which varies based on the vertical structure on the ocean. It is density following (isopycnic) in the stratified ocean, pressure following (level) in the surface mixed layer and terrain-following (sigma) in coastal areas and on shelf seas.

Connectivity matrices were generated for the 8km global HYCOM driven dispersal simulation, as well as for the 4km regional Hawaiʻi HYCOM driven simulation. For the full archipelago simulations we used the habitat definitions in the main manuscript and released 50 particles from 687 sites each day for five years (May 1, 2009-May 2014). For the MHI domain we used a subset of the coral reef habitat for the smaller geographic domain, releasing 50 particles from each of the 406 habitat pixels every day from May 2, 2009-May 25, 2014.

Forward matrices report the final locations of particles released at a given island, as measured by the percent of successfully settled particles released from the source site (*i*) that “settled” at the receiving site (*j*) and can be written in the following equation:

Pij (forward) = Sij/sum Si

Rearward probability matrices report origin sites of particles arriving at the receiving site and can be written

Pij (rearward) = Sij/sum Sj

**Results**

Archipelago wide simulation yielded similar probability matrixes for model runs using 0.08 HYCOM (Fig S4) and regional MITgcm, despite having different resolutions. The global HYCOM for the Hawaii region showed a greater connectivity between more distant islands than did MITgcm. Johnston atoll showed consistently high levels of self-recruitment (close to 100%) with no discernible influx of propagules from Hawai‘i.

*Difference Matrices for the Hawaiian Archipelago*

Comparing the differences between transport model runs using 0.08° HYCOM vs. regional (0.04°) MITgcm currents, the rearward probability matrix (Fig S5b) show a stronger correlation (r=0.9259) than the forward matrix (Fig. S5a) (r=0.9123). Within the matrices, the forward transport probabilities are more different in the MHI indicated by the larger values in the difference matrix (Fig S5a), whereas rearward probabilities show greater differences in the NWHI. In the forward matrix, 0.08° HYCOM overestimates probabilities for the whole archipelago, except for the center of the archipelago where MITgcm shows higher probabilities or transport. This is most apparent around St. Rogatien and its banks, but also in Moloka‘i, and somewhat around Laysan and Raita. In the rearward matrix (Fig. S5b), 0.08° HYCOM show higher probability between more distant locations, and show distant connections that are not present in the regional MITgcm runs.

*Difference Matrices for the Main Hawaiian Islands*

The forward potential connectivity matrix comparing MITgcm and 0.04° HYCOM for the Main Hawaiian Islands (MHI) are most similar (r=0.9102) (Fig S7 A1). Curiously, 0.08° HYCOM and 0.04° MITgcm forward matrices (r=0.8584) (Fig S7 B1) are slightly more similar than the 0.08° and 0.04° HYCOM (r=0.8315) (Fig S7C1). The 0.08° HYCOM seems to overestimate connectivity compared to 0.04° HYCOM and 0.04° MITgcm throughout the MHI, except for Moloka‘i and Lāna‘i in the 0.04° HYCOM comparison.

Rearward connectivity differs in patterns of correlation strength between models compared to forward connectivity. Rearward difference matrices show that the two resolutions of HYCOM have the strongest correlation (r=0.974)(Fig S7C2). The 0.04° MITgcm and 0.04° HYCOM show slightly less agreement (r=0.9533) (Fig S7A2) than 0.08° HYCOM and 0.04° MITgcm (r=0.9305) (Fig S7B2). Disagreements between models in rearward matrices vary more than the forward matrices; there is not one model that overestimates potential connectivity for the whole MHI.

References:

[Bleck, Rainer. 2002. “An Oceanic General Circulation Model Framed in Hybrid Isopycnic-Cartesian Coordinates.” *Ocean Modelling* 4 (1). Elsevier: 55–88.](http://paperpile.com/b/xE5HWy/ftvD)

[Chassignet, Eric, Harley Hurlburt, E. Joseph Metzger, Ole Smedstad, James Cummings, George Halliwell, Rainer Bleck, et al. 2009. “US GODAE: Global Ocean Prediction with the HYbrid Coordinate Ocean Model (HYCOM).” *Oceanography*  22 (2): 64–75.](http://paperpile.com/b/xE5HWy/JYyD)
